# Transmission dynamics for invasive Non-Typhoidal *S* *almonella* serovars (TiNTS): protocol for a household study of transmission and immune response to non-typhoidal *Salmonella* in Malawi

**DOI:** 10.12688/wellcomeopenres.24663.1

**Published:** 2025-10-16

**Authors:** Peter I. Johnston, Kenneth Chizani, Esmeda Chirwa, Helen Dale, Pyianka Patel, Niza Silungwe, Chifundo Mkwangwanya, Tamando Kachala, Chipiliro Mhango, Grace Nyirenda, Yohane Diness, Star Mpesi, Richard Wachepa, Florence Shumba, Felistas Mwakiseghile, Vincent Rashid, Theresa Misiri, Philip M. Ashton, Angeziwa Chunga, Derek Cocker, Edward Cunningham-Oakes, Chris Jewell, Nicholas Feasey, Melita A. Gordon, Tonney Nyirenda

**Affiliations:** 1 Clinical Infection, Microbiology and Immunology, University of Liverpool Institute of Infection Veterinary and Ecological Sciences, Neston, England, UK; 2 Malawi Liverpool Wellcome Clinical Research Programme, Blantyre, Malawi; 3 Kamuzu University of Health Sciences, Blantyre, Southern Region, Malawi; 4 Centre for Genomic Pathogen Surveillance, University of Oxford, Oxford, England, UK; 5 Department of Microbes, Infection and Microbiomes, School of Infection, Inflammation and Immunology, College of Medicine and Health, University of Birmingham, Birmingham, England, UK; 6 Institute of Microbiology and Infection, University of Birmingham School of Biosciences, Birmingham, England, UK; 7 School of Mathematical Sciences, Lancaster University, Lancaster, LA1 4YF, UK; 8 University of St Andrews Faculty of Medicine, St Andrews, Scotland, UK; 9 The University of Edinburgh Usher Institute of Population Health Sciences and Informatics, Edinburgh, Scotland, UK

**Keywords:** Non-typhoidal Salmonella (NTS), transmission, genomics, metagenomics, longitudinal cohort study, Environmental reservoirs, antimicrobial resistance (AMR), Urban informal settlement

## Abstract

**Background:**

Invasive non-typhoidal *Salmonella* (iNTS) disease is a leading cause of community-onset bloodstream infection in Africa, driving high morbidity in young children. The World Health Organization has published preferred product characteristics for an iNTS vaccine, but lack of transmission data is an impediment to vaccine licensure. Enteric NTS (eNTS) is the asymptomatic carriage of NTS in stool that precedes invasive disease. We do not know how long eNTS shedding lasts, how often infection spreads in endemic settings, or how an eNTS episode shapes immunity against later invasion. These gaps make it difficult to define trial sites, select cohorts, refine target product profiles, and build reliable models of vaccine impact. Here we describe TiNTS, a prospective household study in Blantyre, Malawi, which will measure real-time eNTS incidence, transmission, and antibody responses to close these evidence gaps and accelerate rational vaccine deployment.

**Methods:**

We will recruit all members of at least 60 households in Ndirande, Blantyre, Malawi. Stool samples will be collected every other day for at least four weeks and tested for NTS using culture and pan- *Salmonella* PCR on growth media. Environmental samples collected at enrolment will be tested using the same methods. Symptoms and exposure risks will be recorded throughout.

We will collect blood samples at enrolment, after four weeks, and four weeks after the first eNTS episode in each household. We will measure serum IgG responses to *Salmonella* Typhimurium and Enteritidis LPS antigens. We will extend follow-up if participants continue shedding or if the first household case occurs with fewer than 14 days of follow-up remaining.

All culture-positive isolates and PCR-positive broths will undergo Illumina sequencing to enable genome and metagenome reconstruction for transmission inference.

**Conclusions:**

TiNTS will define the burden, transmission patterns, and immune response to eNTS. Findings will inform vaccine modelling, trial design, and targeted introduction strategies.

## Plain Language Summary

Non-typhoidal salmonellae (NTS) are bacteria that are spread by the faecal-oral route. Once in someone’s gut, they can do one of three things: 1) cause no symptoms (termed “enteric NTS”, or “eNTS”) 2) cause diarrhoea (termed “diarrhoeal NTS”, or “dNTS” 3) escape from the gut and cause infection in the bloodstream (termed “invasive NTS” or “iNTS”). Many who develop iNTS disease will die.

In Africa, iNTS disease is common. This is because NTS bacteria have adapted to escape immune defences in the gut, and because illnesses that dampen the body’s defence against the bacteria, such as malaria, malnutrition, anaemia, and HIV, are frequent.

In order to prevent iNTS disease, we need to understand how the bacteria that cause it are circulating in communities. How long are individuals infectious? How many people can they spread infection to? What factors predispose to eNTS infection? It is also important to understand the degree to which eNTS can protect against iNTS disease by stimulating natural immunity.

Our study will track entire households over at least four weeks, in a setting where NTS bacteria are known to be circulating in the community, and where iNTS disease is seen in local hospitals. We will see how often members of the household experience eNTS infection, and whether they pass it on to their household contacts. We will find out how long individuals are shedding the bacteria in their stool (the period for which they are infectious). We will look for NTS bacteria in the water supply and household environment. We will find out what effect an episode of eNTS has on a participants’ immune response to NTS bacteria. This information will help to estimate whether vaccines and other interventions might reduce the burden of iNTS disease.

## Introduction

### Background and rationale

Invasive non-typhoidal *Salmonella* (iNTS) disease is a disseminated bacterial infection that causes over 500,000 cases and 4.7 million DALYs annually, with the greatest burden in children living in Africa South of the Sahara ^1– 4^. The vast majority of cases are caused by *Salmonella enteritidis* subspecies *enterica* serovars Enteritidis and Typhimurium ( *Salmonella* Enteritidis and Typhimurium, respectively) ^3^. These serovars are genetically predisposed toward extra-intestinal disease ^5– 9^. The World Health Organization (WHO) has identified iNTS disease as a priority target for vaccine development ^10^ and produced preferred product characteristics for a vaccine protecting against both serovars ^11, 12^.

Understanding the transmission dynamics of bacteria causing iNTS disease is critical to advancing vaccine development and informing public health strategies. Enteric NTS (eNTS), defined as short-term intestinal carriage without gastroenteritis, is a necessary precursor to invasive disease and distinct from diarrhoeal NTS (dNTS) ^13^. Although gastrointestinal salmonellosis is a globally important foodborne illness, it is essential not to conflate sporadic foodborne outbreaks in high-income settings with the sustained environmental exposure that underpins iNTS transmission in endemic regions.

Recent expert consultations have highlighted critical knowledge gaps, including the need for detailed epidemiological surveillance of both invasive and enteric NTS infections, identification of transmission pathways and environmental reservoirs, clarification of risk factors influencing asymptomatic carriage and immunity, and granular, age-stratified data to inform vaccine scheduling ^11^. These data are vital to accurately model vaccine impact across diverse populations, particularly in settings with high antimicrobial resistance and prevalent comorbidities such as HIV and malaria. Addressing these gaps will support vaccine licensure, strategic deployment, and prioritisation efforts for iNTS vaccines in Africa.

Here we describe the TiNTS ( **T** ransmission dynamics for **i** nvasive **N** on- **T** yphoidal ***S*** *almonella* serovars) study, which directly addresses these research priorities through prospective, longitudinal household sampling combined with advanced genomic and modelling methodologies.

### Broad objectives

1. Estimate key parameters of NTS transmission in an endemic setting, including incidence, prevalence, shedding duration, and secondary attack rates.
2. Quantify how gastrointestinal NTS infection influences the development of antibody responses to iNTS serovars.
3. Identify individual- and household-level risk factors for NTS acquisition and transmission.

### Specific objectives

1. Estimate the incidence and prevalence of eNTS using both culture and PCR-based detection methods.
2. Measure the duration of NTS shedding using culture, PCR, and genomic methods.
3. Reconstruct transmission pathways using genomic and metagenomic data from participants and the household environment.
4. Estimate key transmission parameters such as secondary attack rates, shedding duration, and environmental persistence.
5. Quantify the impact of eNTS episodes on antibody acquisition to *Salmonella* O:4 and O:9 LPS antigens.
6. Identify individual- and household-level factors associated with increased risk of eNTS.
7. Characterise the symptoms of gastrointestinal NTS and estimate the proportion of asymptomatic versus symptomatic cases.
8. Build and calibrate a transmission model using study data to assess the likely impact of vaccination and public health interventions.

## Methods

### Study design overview

TiNTS is a prospective, community-based cohort study designed to enrol and longitudinally follow at least 60 entire households. Stool cultures will be collected every other day for at least 28 days to detect *Salmonella* colonisation, and blood samples will be obtained at enrolment, 28 days later, and 28 days after the first eNTS episode in the household. At enrolment, we will also conduct environmental sampling and complete both individual and household-level case record forms. The study includes procedures to capture prolonged stool shedding and transmission events occurring late in follow-up. For a breakdown of all study visits please refer to Table 1 and the [Study procedures] section.

**Table 1.** Overview of study visits, their purpose, and study activities conducted at each visit.

Visit	Timing	Participants	Samples/Activities	Purpose
**Visit 1 (enrolment)**	Day 0	All household members	Consent/assent Administer CRFs (individual and household) Blood sample Stool sample Nasopharyngeal swab Environmental samples: (water, bootsock, Moore swab)	Participant enrolment Baseline data and specimen collection
**Routine visits**	Every other day (14 visits in 28 days)	All household members	Symptom screening Stool sample collection Blood sample (on day 28 only)	Detection of eNTS carriage and symptom monitoring Assess change in antibody titre from baseline (day 28 visit only)
**Triggered follow-up visit**	28 days after first positive stool culture	All household members	Blood sample Symptom/exposure CRF	Assess immune response following eNTS exposure
**Extended follow-up**	Day 29+ (if culture positive detected after Day 14)	All household members	Continued stool sample collection Symptom screening	Ensure 14-day window post-index case for transmission
**Prolonged shedding follow-up**	Weekly until two negative samples	Participants shedding at Day 28+	Stool sample Symptom screening	Measure duration of shedding

### Study setting

TiNTS will be conducted in Ndirande, Malawi. Ndirande is an informal settlement characterised by poor water, sanitation, and hygiene (WASH) infrastructure and high population density. 97,839 inhabitants were recorded in the 2018 census ^14^. The Malawi Liverpool Wellcome Clinical Research Programme (MLW) has longstanding links to the community of Ndirande, and maintains a permanent presence at Ndirande Health Centre.

**Figure 1.**
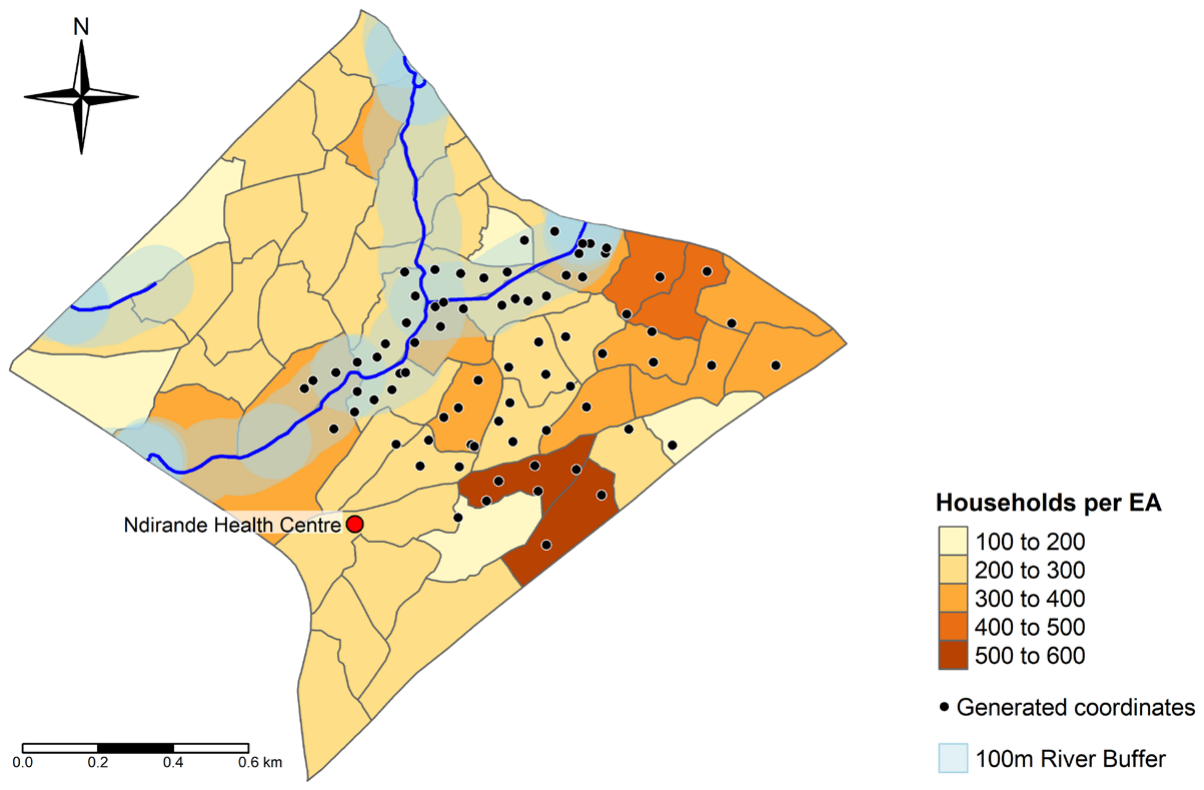
Map of Ndirande, showing the randomly generated co-ordinates from which households will be selected for the study. The Nasolo river is shown with a 100 metre buffer zone surrounding it. Grey borders represent enumeration areas (EA) from the 2018 Malawi census, and the density of households within each of these is represented by colour as detailed in the legend. The location of Ndirande Health Centre is shown as a red circle.

To define our sampling framework, we obtained the shapefile for Ndirande from 2018 census data, and overlaid this with river water data from OpenStreetMap ^15^. We used the tmap (V3.4-0) ^16^, osmdata (V0.2.2) ^17^, and sf (1.0-15) ^18^ packages in R (V4.3.3) ^19^ to overlay randomly generated co-ordinates within the bounds of Ndirande: half of the co-ordinates were generated within a 100 metre buffer zone of the Nasolo river, which bisects Ndirande, whilst the remainder were distributed outside this buffer zone. We used this method to enrich for NTS carriage through proximity to a major waterway. To allow study personnel to move on foot, we restricted sampling to enumeration areas (EAs) in the South-East corner of Ndirande, which are readily accessible from Ndirande Health Centre. The study site and sampling co-ordinates are shown in Figure 1.

### Eligibility criteria

**Figure 2.**
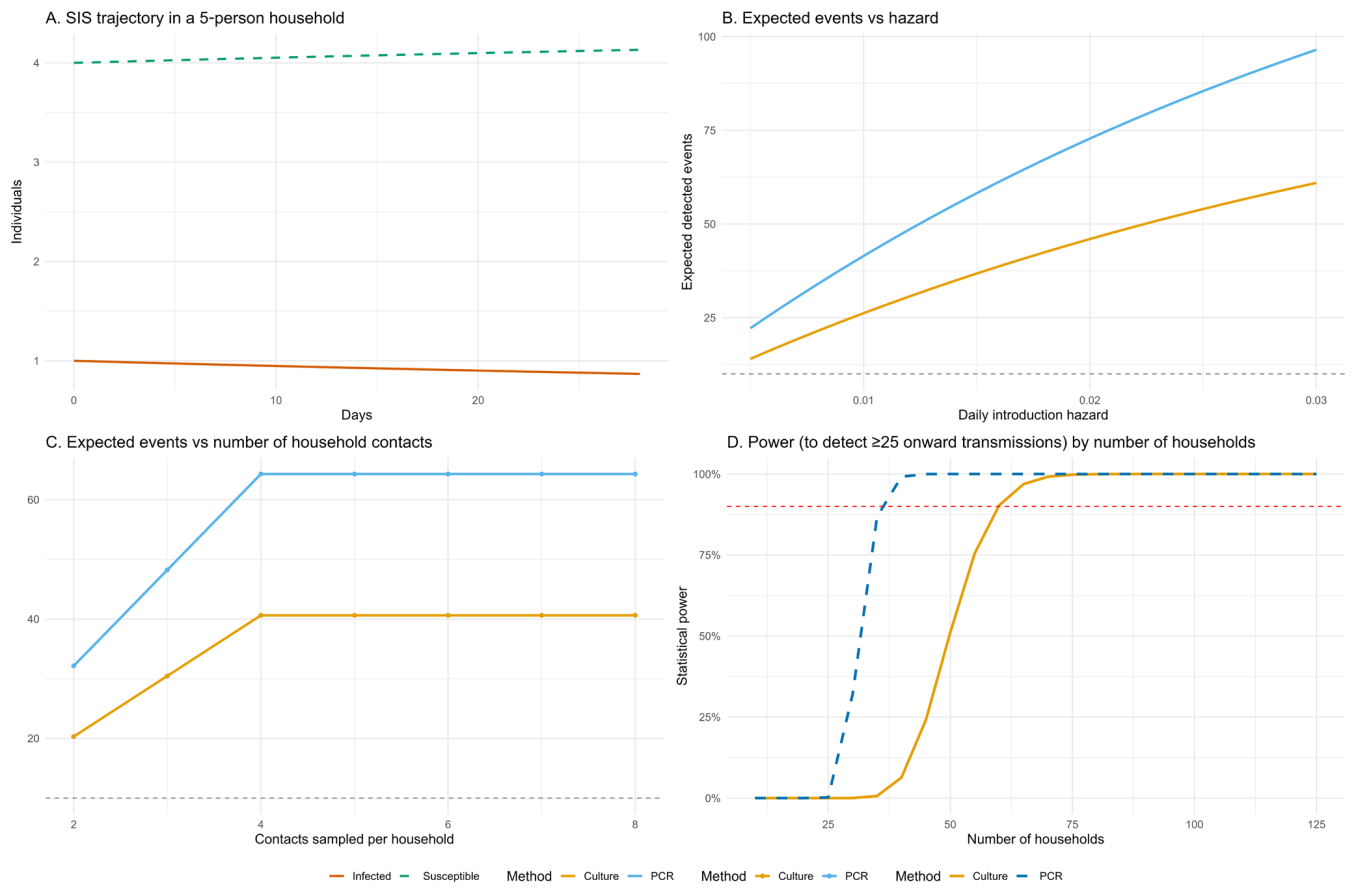
Simulation-based estimation of expected events and statistical power for within-household Salmonella transmission. Panel **A** shows SIS model dynamics over 28 days in a 5-person household with one initial infection. Panel **B** plots the expected number of detected events in 60 households across a range of daily introduction hazards. Panel **C** shows the effect of increasing the number of contacts sampled per household on expected detected events, assuming a fixed hazard of 0.0115. Panel **D** displays statistical power to detect ≥25 onward transmission events, plotted against increasing household sample size, with a 90% power threshold indicated (red dashed line). Results are shown for both PCR and culture-based detection methods, incorporating sensitivity estimates from Chirambo *et al.* (2021) ^21^.

We will recruit participants of all ages residing in eligible households within our study site. We will only recruit households within which four or more people reside permanently. This is because our modelling predicts that transmission will be less frequently detected in small households (see Figure 2, Panel C). We will only enrol households where at least one child resides. This is to ensure that our findings are generalizable to young children, who carry the greatest burden of iNTS disease ^1^, and are therefore likely to be the target population for vaccination ^11^.

Individuals of any age will be eligible, but children must have a responsible adult able to provide informed consent (assent forms will be completed in addition, for children aged between 7 and 17 years). We will not include households who intend to move away from their current residence within three months of enrolment.

### Sample size and power calculation

We developed a simple SIS (Susceptible-Infected-Susceptible) model which showed that at least 60 households should be recruited in order to detect 25 within-household transmission events (using stool culture) with 90% power ( Figure 2, panel D). Our model simulated a five person household followed over 28 days, starting with one index eNTS case. We parameterised this model using a mean infectious period of 28 days, which we derived from the limited evidence pertaining to duration of NTS shedding currently available in the literature ^20^. We used a prevalence estimate of 5.25%, based on an estimate derived from a longitudinal study of healthy children recruited from a vaccine clinic at Zingwangwa health centre, which serves an urban population close to our own study site ^21^. Using the model, we estimated the transmission rate (β) under endemic equilibrium and used this to generate expected infection trajectories. From these, we calculated the average number of infected contacts over time.

We combined this with evidence-based assumptions about diagnostic test sensitivity (62.9% for stool culture and 99.5% for PCR ^21^) and the number of household contacts sampled to estimate the expected number of detected onward transmission events. We defined an event as an additional infection within a household where one individual had already been infected. We reasoned that although some cases may represent separate introductions, enrolling 25 households would likely capture at least ten genuine transmission events. We simulated event detection under a range of daily introduction hazards and sampling scenarios, assuming households were culture-negative at baseline ( Figure 2, Panel B). Finally, we calculated the number of households required to detect a minimum threshold of 25 events with 90% probability, using a conservative daily introduction hazard of 0.0115 within a binomial model for both culture and PCR positive events. ( Figure 2, Panel D).

All simulations and plots were generated in R (version 4.3.3) ^19^ using the packages deSolve (1.40) ^22^, ggplot2 (3.5.1) ^23^, dplyr (1.1.4) ^24^, scales (1.3.0) ^25^, and patchwork (1.3.0) ^26^ All R code and simulation outputs used for the power calculations are available via Zenodo: https://doi.org/10.5281/zenodo.15675205.

### Household selection and recruitment

We will select households using a Geographic Information System (GIS)-based bottle-spin method. At each randomly generated coordinate, field staff will spin a bottle to determine a direction, then approach the first household encountered along that path. If the household declines to take part or is ineligible, we will return to the original point and repeat the process until we identify an eligible and willing household.

Our study team will explain the study and seek written informed consent and assent (from minors aged seven to 17 years) as appropriate.

### Interventions and participant monitoring

This is a non-interventional observational study. We will not provide antibiotic treatment to asymptomatic participants from whom we grow *Salmonella*, as most infections resolve spontaneously, and there is no evidence to suggest clinical benefit from antibiotics ^27^. Qualified nurses will closely monitor participants and refer anyone who becomes unwell to appropriate health services.

### Study procedures

**Figure 3.**
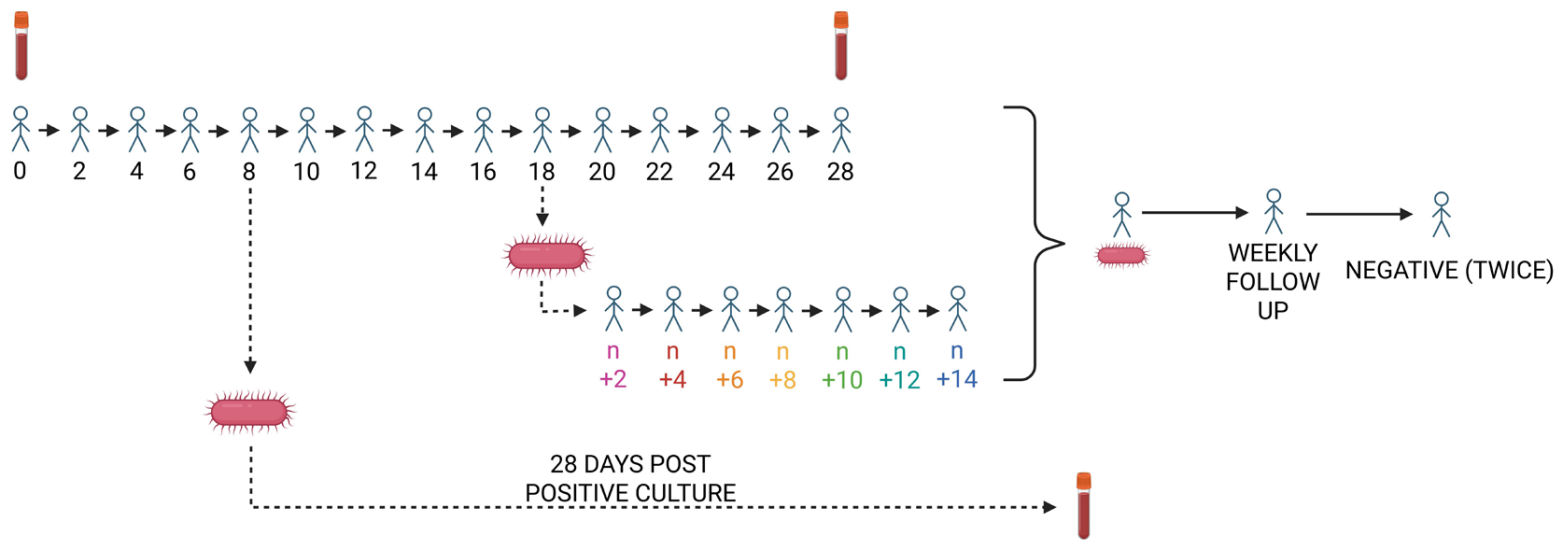
Overview of TiNTS study visits. Households are enrolled at day zero and followed through day 28. When Salmonella is first cultured from a participant's stool, an additional visit is scheduled for 28 days after the positive stool sample date (illustrated by the arrow from day 8). If the first positive culture occurs with fewer than 14 days remaining in the routine follow-up, visits are extended to ensure 14 days of monitoring after the positive result (illustrated by the downward arrow from day 18). Participants who remain stool culture-positive at the end of household follow-up undergo weekly stool sampling until two consecutive samples are negative (arrows at right). Blood draws for antibody measurements are indicated by vacutainer tubes.

***Study visits.*** An overview of study visits is provided in Table 1 and a schematic illustration is shown in Figure 3. Each household will be visited a minimum of 15 times: one enrolment visit followed by 14 visits for routine stool sampling. Stool cultures will be processed in real-time to inform future visit schedules.

If no *Salmonella* is cultured from any household member during follow-up, sampling will conclude after 28 days. If *Salmonella* is detected in stool, we will record the earliest positive date and conduct an additional blood draw from all household members 28 days later.

If the first positive stool culture occurs with fewer than 14 days of routine follow-up remaining, visits will be extended to ensure a full 14-day observation window after the index case.

If an individual remains culture-positive at the end of the household follow-up, they will continue weekly follow-up until two consecutive negative stool samples are obtained, allowing us to estimate shedding duration.

### Data collection tools

***Household and individual level Case Record Forms.*** At enrolment, each participant will complete an individual case record form (CRF) with research personnel, who will record answers on an encrypted tablet device. CRF questions will be available in English and Chichewa languages. Guardians will assist minors in completing their CRFs.

We will also complete a household-level CRF, with responses provided by one or more participants.

CRFs will capture demographics, health details, symptom data, eNTS risk factors, wealth and socioeconomic indicators, and contact and social mixing patterns. Our CRFs take inspiration from those developed by the DRUM consortium, which were designed by an interdisciplinary working group as previously described ^28^.

A brief CRF recording symptom data will be administered at every study visit. Participants who test positive for *Salmonella* in stool will complete a more comprehensive CRF capturing symptoms and potential exposure risks at their next visit. Complete versions of the CRFs (in English and Chichewa) are available on **Zenodo: https://doi.org/10.5281/zenodo.15690901**.

### Biological sample collection

***Stool collection.*** We will collect stool samples from every participant at each visit until day 28. At every visit, we will provide sterile stool pots and biodegradable bed pans. Research nurses will demonstrate how to partially fill these pots using a spoon attached to the lid. If participants under five years cannot produce stool, nurses will offer a rectal swab (where there is parental consent).

***Blood draws.*** Research nurses will draw blood from all participants at the enrolment visit, 28 days after enrolment, and 28 days after the first stool sample that was culture-positive for *Salmonella* was collected (where applicable). We will limit blood volumes to a maximum of 2 mL/kg for children under 7 kg. We will collect 5ml blood from all other participants ^29^.

***Nasopharyngeal swabs.*** Research nurses will swab the nasopharynx of each participant at the enrolment visit in order to detect whether NTS bacteria may be harboured.

***Environmental samples.*** Environmental samples will be collected at the enrolment visit, with the exception of Moore swabs, which will be deployed at the enrolment visit and retrieved 48 hours later.

Three samples of water will be collected from each household in one litre receptacles. Drinking water will be prioritised, and where no water is stored, a sample will be taken from the household’s drinking water source, such as a shared borehole or tap.

Three bootsock samples will be collected per household: study personnel will don sterile overshoes and walk ten steps within the household environment, targeting areas such as kitchens and latrines.

Where households access river water routinely, a Moore swab will be placed close to the access point ^30^. The Moore swabs will be made by the field team and autoclaved in the laboratory before deployment.

***Sample transport.*** Samples will be placed in labelled specimen bags and placed in a cool box whilst study procedures are completed. They will be brought to the lab and processed on the same day.

Standard operating procedures for all field procedures are available at **Zenodo: https://doi.org/10.5281/zenodo.15690901**

### Laboratory methods

We will process stool, rectal swabs, nasopharyngeal swabs, bootsock samples, Moore swabs, and household water grab samples to isolate *Salmonella* species and store them for downstream genomic and immunologic analyses. We will store isolates at –70°C for later sequencing. All procedures will follow standardised protocols which have been developed and approved by the Malawi Liverpool Wellcome Trust. All laboratory protocols have been deposited online at Zenodo and are available at **Zenodo: https://doi.org/10.5281/zenodo.15690901**

**Figure 4.**
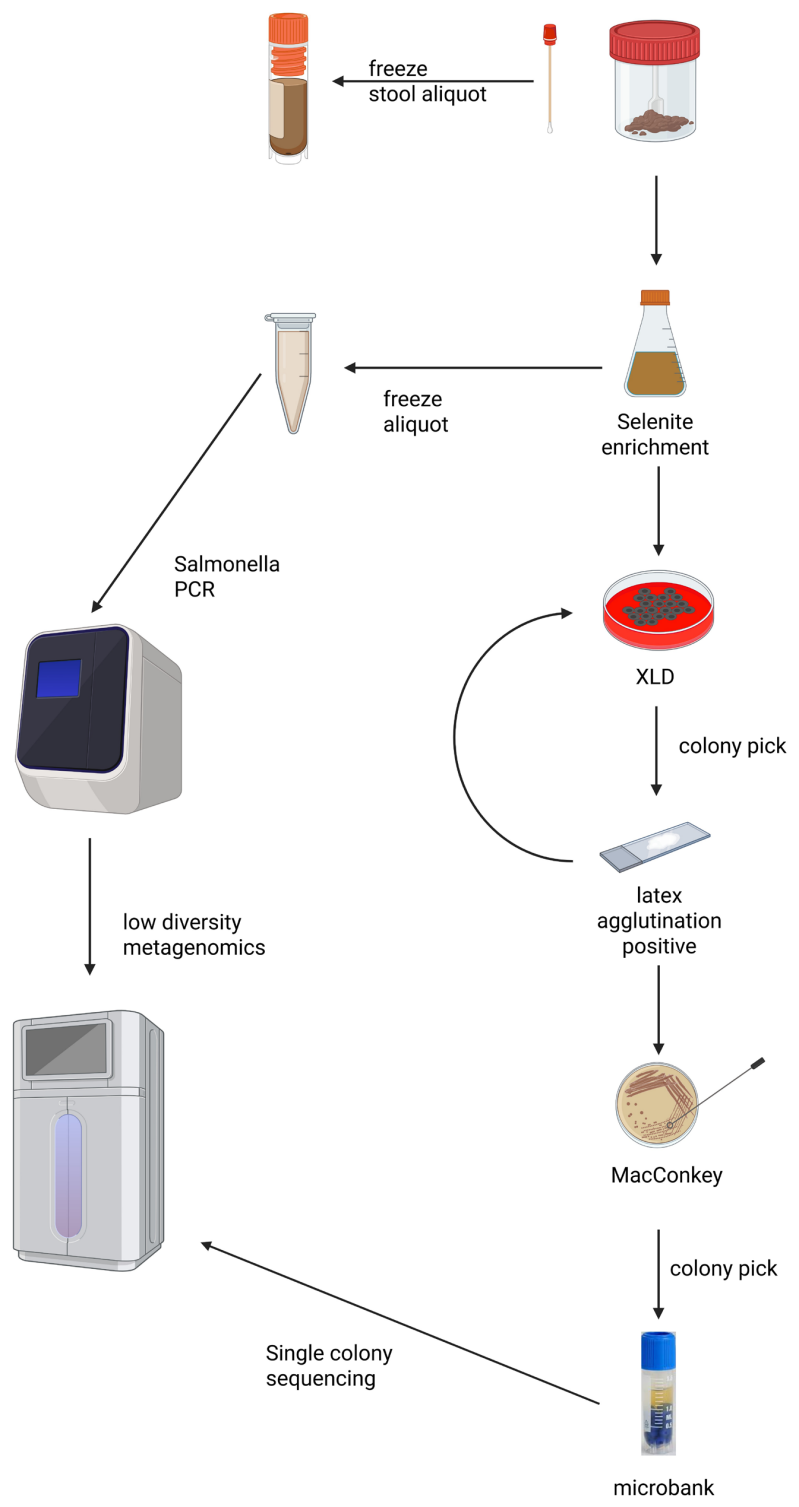
Overview of laboratory procedures applied to stool samples. The diagram outlines selective enrichment and culture steps for Salmonella detection, PCR screening, archiving of isolates, and the downstream selection of samples for whole-genome sequencing (single colonies) or low-diversity metagenomic sequencing (PCR-positive enriched media).

***Stool and rectal swabs.*** On receipt, we will aliquot stool samples, freezing one portion and inoculating the other into both selenite broth and buffered peptone water (BPW). For rectal swabs, we will inoculate directly into both broths. After overnight aerobic incubation, we will freeze aliquots and centrifuge the selenite broth. We will inoculate the resulting sediment onto XLD agar and incubate it overnight. We will identify presumptive *Salmonella* colonies based on morphology and confirm them using a *Salmonella* latex agglutination test (Oxoid, ThermoFisher DR1108A). We will assess antigenic expression using O:4 and O:9 antisera. We will sub-culture confirmed isolates onto MacConkey agar and cryopreserve single colonies and plate sweeps in microbank vials. Please refer to Figure 4 for an overview of microbiologic methods.

***Nasopharyngeal swabs.*** We will inoculate swabs into selenite broth, incubate them overnight, and process them identically to stool samples, starting from the centrifugation and plating stage.

***Bootsock samples.*** We will incubate each sock in BPW overnight, then transfer 1 mL of the broth to selenite broth for selective enrichment. We will then process the sample using the same culture, confirmation, and storage steps as for stool.

***Water grab samples.*** We will filter up to 500 mL of each water sample using 0.45 µm membrane filters. We will incubate each filter in BPW overnight, transfer 1 mL to selenite broth, and proceed with enrichment, plating, and identification as described above. These methods have been previously optimised for the detection of *Salmonella* Typhi in our setting ^31^.

***Moore swabs.*** After retrieval from rivers, we will incubate Moore swabs in BPW and process them in the same way as water grab samples.

***Serum.*** We will centrifuge blood samples within three hours of collection. We will aliquot serum into four labelled cryovials (three × 300 µL and one remainder aliquot), generate a cryobox map, and store samples at –70°C. These samples will be used for ELISA-based measurement of anti-LPS antibody titres and other immunological assays.

***ELISA for Anti-Lipopolysaccharide Antibodies.*** We will quantify serum IgG responses to *Salmonella* lipopolysaccharide (LPS) using a standard indirect ELISA. We will coat high-binding 96-well plates with purified LPS from *Salmonella* Typhimurium or *Salmonella* Enteritidis (Enzo Life Sciences) and incubate them overnight at 4°C or for 1 hour at 37°C. Following washing, we will block the plates using a buffered protein-based blocking solution to reduce non-specific binding.

Test serum will be thawed on ice and added in serial 3-fold dilutions starting at 1:20. We will incubate the plates at 37°C to allow binding of LPS-specific antibodies, followed by a wash step and the addition of alkaline phosphatase-conjugated anti-human IgG secondary antibody. After a further incubation and wash, we will add phosphatase substrate and measure absorbance at 405 nm using a plate reader.

We will determine antibody responses by fitting dilution curves to calculate log₂ endpoint titres and EC50 values using four-parameter logistic regression. We will include internal controls and replicate wells to assess assay reproducibility.

***Identification and storage.*** For all sample types, we will identify presumptive *Salmonella* using latex agglutination and antisera testing. We will sub-culture confirmed isolates on MacConkey agar and store both colony picks and plate sweeps in microbank tubes.

***PCR testing.*** We will test all enriched selenite and buffered peptone water (BPW) broths for the presence of *Salmonella* DNA using a real-time PCR assay targeting the *ttr* gene. Prior validation work at MLW has shown that this target is highly specific and sensitive for pan- *Salmonella* detection when used after pre-enrichment ^21^. We will extract DNA robotically using the KingFisher™ Flex platform with magnetic bead-based purification, following the MagMAX™ Pathogen RNA/DNA protocol. For each sample, we will extract from 200 µL of enriched broth and elute into 90 µL of DNA, using 5 µL as input per PCR reaction.

We will perform PCR in 96-well plates on the Applied Biosystems™ 7500 Real-Time PCR System. Each run will include a positive control derived from *Salmonella* Typhimurium strain D23580 and a no-template control. We will monitor amplification curves and apply an automatic threshold within the ABI software. We will classify samples with a sigmoid curve and a Ct value below a predetermined cutoff as positive.

To determine the appropriate Ct threshold for positivity, we will use our own whole genome sequencing data from single colony *Salmonella* isolates as the gold standard. We will fit a Bayesian latent class model to estimate the probability that a given PCR result reflects true infection, accounting for uncertainty in both culture and PCR performance. This will allow us to define an optimal Ct cutoff that maximises detection while controlling for false positives, informed by the empirical distribution of Ct values in sequence-confirmed samples.

***Genomic and Metagenomic Sequencing.*** We will perform Illumina whole-genome sequencing on all archived *Salmonella* isolates derived from single colony picks. In parallel, we will subject PCR-positive enriched growth media to deep Illumina sequencing to enable recovery of *Salmonella* genomes in the context of low-diversity microbial communities. This combined approach will support high-resolution strain typing, antimicrobial resistance profiling, and transmission inference.

### Statistical and bioinformatic analysis

We will take an integrated statistical and genomic approach to characterising *Salmonella* carriage, transmission, and immune response in the household setting. Our analyses will include descriptive epidemiology, latent infection modelling, survival analysis, transmission dynamics, risk factor evaluation, and genome-informed inference.

***Covariate derivation and household wealth index.*** We will build a composite household wealth index using polychoric principal component analysis. This method combines binary and ordinal indicators of socioeconomic status into a single continuous variable. It captures both structural and material differences between households. We will use this index as a covariate in all models. It will help adjust for confounding, especially in settings where poverty, sanitation, and exposure to *Salmonella* overlap.

***PCR classification and latent infection modelling.*** We will use a subset of bioinformatically confirmed *Salmonella* isolates as the reference standard to evaluate PCR performance. By linking Ct values from PCR to culture and genomic confirmation status, we will fit Bayesian latent class models to estimate the probability that a given PCR result represents true infection. This approach allows us to define a data-driven Ct threshold and account for misclassification across the full dataset.

***Descriptive epidemiology and shedding dynamics.*** We will summarise *Salmonella* carriage by calculating incidence, prevalence, and shedding duration at individual and household levels. Shedding duration will be defined from the first to last PCR-positive stool sample. We will visualise clearance using Kaplan–Meier survival curves and fit Cox proportional hazards models to identify factors associated with prolonged shedding. All models will include shared frailty terms to account for within-household clustering, whilst acknowledging that serial dependence in individual hazard rates due to autochthonous transmission might still remain (and thus motivating the transmission modelling approach below).

***Risk factor analysis.*** We will explore predictors of *Salmonella* acquisition using multivariable Cox models. Covariates will come from individual and household CRFs. We will first reduce dimensionality through expert review. We will then fit Bayesian Cox models using shrinkage priors. All models will include household-level frailty terms to adjust for shared exposure. As above, the approach assumes independence between individuals, which may not hold in households with active transmission. This further motivates our explicit modelling of transmission.

***Transmission modelling and secondary attack rates.*** We will estimate how often *Salmonella* spreads from one household member to another, or secondary attack rate. We will model the risk of acquisition based on contact with infected individuals over time. These models will adjust for covariates and account for the fact that one person’s infection increases another’s risk. We will add genetic data to strengthen our inferences. This includes whole genome sequencing and metagenomic reconstruction. Where isolates match, we will interpret this as support for direct transmission. Together, this approach gives us power to measure who infected whom, and when.

***Genomic and metagenomic inference.*** We will perform Illumina whole genome sequencing on all single colony isolates. For samples that are PCR-positive but culture-negative, we will apply deep Illumina sequencing of enriched growth media to recover *Salmonella* genomes using low-diversity metagenomic techniques. We will use core genome alignments and SNP distance matrices to infer relatedness between isolates, identify likely transmission clusters, and evaluate strain persistence within households. Metagenomic data will also be used to assess co-colonising species and potential microbial competitors or facilitators.

***Serological analysis and immunological inference.*** We will quantify serum IgG responses to lipopolysaccharide antigens from *Salmonella* Typhimurium and Enteritidis. We hypothesise that these responses may arise from exposure to both invasive and non-invasive strains, including environmental serovars that share the same O-antigen groups. We will compare antibody titres before and after documented episodes of *Salmonella* detection and assess whether immune priming follows subclinical colonisation. We will also examine baseline titres as a potential marker of prior exposure and correlate these with future carriage or protection.

This combined statistical and bioinformatic framework will allow us to reconstruct *Salmonella* transmission, estimate infection probabilities, identify modifiable risk factors, and understand how immune responses are shaped by both clinical and environmental exposure.

***Missing data.*** Not all participants will be present at all scheduled study visits. For serological analysis, participants must have at least two antibody measurements at separate timepoints to be included in immune response modelling. If a participant is found to be shedding *Salmonella* in stool but misses subsequent sampling points, we will make rational, case-by-case decisions regarding the estimated duration of shedding. This will be informed by the timing and genetic similarity of subsequent positive samples from the same household.

## Conclusions

TiNTS is designed to generate the high-resolution data needed to understand how non-typhoidal *Salmonella* spreads within households and communities in an endemic setting. By integrating intensive longitudinal sampling, genomic and metagenomic sequencing, and immune profiling, the study will provide direct estimates of key transmission parameters, shedding duration, and immune correlates following asymptomatic carriage. This evidence will support the refinement of transmission models, help define correlates of protection, and inform the design, testing, and deployment of non-typhoidal *Salmonella* vaccines in high-risk settings.

## Ethics statement

This study has received ethical approval from the University of Liverpool Research Ethics Committee D (ref: 12765) and the College of Medicine Research Ethics Committee (COMREC), Malawi (ref: P.06/23-0093). Sponsorship is provided by the University of Liverpool (ref: UoL001870). All participants (or their guardians, in the case of minors) will provide written informed consent, with age-appropriate assent obtained from children aged 7–17 years. Data will be anonymised prior to analysis and handled in accordance with institutional data governance policies.

## Dissemination

We will share study findings with participants and the wider community in Ndirande through feedback meetings supported by the MLW Science Communication team.

At national level, we will make results available to relevant public health partners, with the aim of contributing to the evidence base for non-typhoidal *Salmonella* vaccine planning in Malawi. We also hope that our data will support broader efforts to inform vaccine licensure through the identification of correlates of protection and improved understanding of transmission dynamics. Findings will be published in open-access journals and presented at relevant scientific meetings.

## Data Availability

Zenodo: *Standard operating / lab / field procedures for Transmission Dynamics for invasive Non-Typhoidal Salmonella Serovars Study*. https://doi.org/10.5281/zenodo.15690901 ^32^. This project contains the following underlying data: analytic_scripts.zip (key analytic scripts for statistical modelling) simulation_code.zip (code for simulation modelling) case_record_forms.pdf (CRFs used for data collection) laboratory_protocols.pdf (protocols for laboratory procedures) Final versions of the dataset used for statistical modelling will be de-identified and shared where ethically permissible. Following study completion, we will deposit all *Salmonella* genome assemblies and raw sequencing data in the European Nucleotide Archive (ENA) under a dedicated BioProject, with accession numbers included in subsequent publications and linked from the main study repository. Zenodo: *Standard operating / lab / field procedures for Transmission Dynamics for invasive Non-Typhoidal Salmonella Serovars Study*. https://doi.org/10.5281/zenodo.15690901 ^32^. This project contains the following extended data: STROBE checklist (completed checklist for reporting observational studies) Data are available under the terms of the Creative Commons Attribution 4.0 International license (CC-BY 4.0). Zenodo: STROBE checklist for *Standard operating / lab / field procedures for Transmission Dynamics for invasive Non-Typhoidal Salmonella Serovars Study*. https://doi.org/10.5281/zenodo.15690901 ^32^. Data are available under the terms of the Creative Commons Attribution 4.0 International license (CC-BY 4.0)
