# Supplementary material for: Transmission dynamics for invasive Non-Typhoidal S almonella serovars (TiNTS): protocol for a household study of transmission and immune response to non-typhoidal Salmonella in Malawi
Source: Wellcome Open Res. 2025 Oct 16;10:581. [Version 1] doi: 10.12688/wellcomeopenres.24663.1 (PMC12635526; doi:10.12688/wellcomeopenres.24663.1)
Supplement: Peer review history

# Reviewer report: Reviewer response for version 1

**DOI:** 10.21956/wellcomeopenres.27178.r137152

**Published:** 2025-12-26

**Authors:** Megan E. Carey

**Affiliations:** 1 London School of Hygiene & Tropical Medicine, London, UK

This protocol describes TiNTS, a prospective longitudinal household study in Ndirande, Blantyre, designed to measure the incidence and duration of enteric NTS (eNTS), reconstruct transmission pathways using genomic and metagenomic methods, and assess antibody responses following exposure. The study rationale is clearly articulated and well aligned with current WHO priorities for iNTS vaccine development. The objectives are logically structured and directly address the major unknowns around eNTS incidence, shedding, transmission, and immunity.

 The intensive household follow-up, high-frequency sampling, and combined genomic and immunological analyses are well suited to the research aims. The overall design is strong and likely to generate the high-resolution data needed for vaccine-relevant insights. The availability of all SOPs, CRFs, and analytic code on Zenodo greatly enhances transparency and reproducibility.

 A few comments/suggestions:

- How will suspected iNTS disease be managed during follow-up? The current text only addresses asymptomatic eNTS.
- Will individuals on antibiotics at enrolment be eligible, given the potential impact on detection and shedding?
- The linked SOP provides household-level inclusion criteria, but a consolidated list of inclusion and exclusion criteria for both households and individuals would improve clarity. In particular, it would help to define whether individuals with acute diarrhoea or febrile illness at baseline, those currently taking antibiotics, temporary household members, or participants who decline certain procedures (e.g., blood draw or nasopharyngeal swab) are eligible for enrolment.
- Please clarify the fallback plan for stool collection in older children and adults who are unable to provide a sample (rectal swabs are currently described only for under-5s).
- Given that young children have the highest incidence and greatest morbidity and mortality associated with iNTS, it would be useful to show how the sampling strategy will yield sufficient numbers of young children. Providing household age-distribution data for Ndirande, and indicating whether enrolment will be monitored or adjusted, would strengthen confidence in this.

Is the study design appropriate for the research question?

Yes

Is the rationale for, and objectives of, the study clearly described?

Yes

Are sufficient details of the methods provided to allow replication by others?

Yes

Are the datasets clearly presented in a useable and accessible format?

Yes

Reviewer Expertise:

infectious disease epidemiology,; genomics; immunisation policy

I confirm that I have read this submission and believe that I have an appropriate level of expertise to confirm that it is of an acceptable scientific standard.

# Reviewer report: Reviewer response for version 1

**DOI:** 10.21956/wellcomeopenres.27178.r137155

**Published:** 2025-11-20

**Authors:** Kelvin Kering

**Affiliations:** 1 Kenya Medical Research Institute, Nairobi, Kenya

In this protocol, the authors present a prospective longitudinal study aimed at investigating the transmission dynamics of Nontyphoidal Salmonella in Blantyre, Malawi. To achieve this objective, all members of at least 60 households will be enrolled and stool samples collected for at least four weeks. In addition, blood and environmental samples will be collected. The stool and environmental samples will be subjected to culture and pan-salmonella PCR, and ultimately, the isolates and PCR broth will be sequenced using Illumina. 

 The collected blood samples will be used to measure serum IgG. A transmission model will also be built and calibrated using the data from the study.

 General comments;

 The protocol is well-written and logical. The study findings will be critical in understanding NTS transmission and inform vaccine use in disease-endemic settings.

 Specific comments

1. What is the average household size in Ndirande? Given that the model predicts less transmission in small households, the average household size in Nidirande should be indicated in the protocol. 
2. Is there a radius limit for each randomly generated coordinate? It would be necessary to indicate the limits of distance that the field staff can cover for every coordinate.
3. Are the authors not interested in detecting invasive NTS disease? What happens if, in the course of follow-up, any household member develops or is confirmed to have iNTS?
4. Will a household member who is on antibiotics on the day of enrollment be included in the study?
5. It is critical that a clear inclusion/exclusion criteria is provided for households. Would anyone with diarrhoea be enrolled? In the eligibility criteria, **households where at least one child resides** will be enrolled, specify the age of the child.
6. Rectal swabs will only be used for under-fives who cannot produce stool, what of adults who, on the day of follow-ups are not able to produce stool? 
7. In case of continuous NTS shedding, would the authors consider ultrasound scans for possible gallstones, given possible NTS colonization of the gallbladder?
8. Antimicrobial resistance of the NTS could be considered in understanding the risk for eNTS.
9. The authors may want to consider three consecutive negative cultures instead of two for participants who remain positive at the end of household follow-up due to the possibility of intermittent shedding.
10. Given the selected household might be some distance from the coordinate used to select the house, the coordinates of the selected household can be collected. Also, consider collecting the GPS coordinates of the Moore swab.
11. Given that the study is being done in an informal settlement, why not consider collecting samples from open drains/children's playgrounds around the household? The household environment is more than the inside of the house.

Is the study design appropriate for the research question?

Yes

Is the rationale for, and objectives of, the study clearly described?

Yes

Are sufficient details of the methods provided to allow replication by others?

Partly

Are the datasets clearly presented in a useable and accessible format?

Not applicable

Reviewer Expertise:

Infectious Diseases, Enteric diseases epidemiology, Nontyphoidal Salmonella

I confirm that I have read this submission and believe that I have an appropriate level of expertise to confirm that it is of an acceptable scientific standard.

# Reviewer report: Reviewer response for version 1

**DOI:** 10.21956/wellcomeopenres.27178.r136548

**Published:** 2025-11-11

**Authors:** Liselotte Hardy

**Affiliations:** 1 Institute of Tropical Medicine, Antwerp, Belgium

Very interesting protocol and study. I have a few small questions, for clarification, but in general everything is clear.

 Interventions and patient monitoring:

 Will there also be no treatment of patients that are suffering from iNTS, if this would be noticed as an adverse event, although this is not tested directly?

 Testing for eNTS:

 - This will be done by culturing and PCR. What data will be used for a final conclusion on the presence of (viable) NTS in the stool?

 - How many colonies will be tested per plate (as there might be variation in the colony types)?

 - How is serotyping done? Is everything dependent on WGS?

Is the study design appropriate for the research question?

Yes

Is the rationale for, and objectives of, the study clearly described?

Yes

Are sufficient details of the methods provided to allow replication by others?

Yes

Are the datasets clearly presented in a useable and accessible format?

Yes

Reviewer Expertise:

Bacterial diagnostics, clinical microbiology

I confirm that I have read this submission and believe that I have an appropriate level of expertise to confirm that it is of an acceptable scientific standard.
